# Finite-Element Analysis of the Quasi-Static Response of Concrete Specimens Containing Polymeric Self-Healing Microcapsules

**DOI:** 10.3390/polym18111289

**Published:** 2026-05-24

**Authors:** Todor Zhelyazov

**Affiliations:** 1 National Institute of Geophysics, Geodesy and Geography, Bulgarian Academy of Sciences, Akad. G. Bonchev str., bl. 3, 1113 Sofia, Bulgaria; elovar@yahoo.com; 2 Structural Engineering and Composites Laboratory (SEL), Reykjavik University, Menntavegur 1, IS-102 Reykjavik, Iceland

**Keywords:** self-healing, polymerization, damage, damage-healing material, concrete, finite element analysis

## Abstract

Healing agent encapsulated in polymeric microcapsules has proven its ability to seal surface and internal cracks. Focused on mitigating the negative impact of capsules on the properties of fresh cement paste and hardened cementitious matrix, uncertainties in self-healing triggering, and poor control of the released quantity, researchers report technological improvements in predominantly experimental studies. However, practical applications will necessitate lightweight models that capture all the characteristics of practical importance. Analysis of the scientific literature reveals the lack of such models adapted for cementitious composites. In this paper, a model rooted in continuum damage mechanics, tuned based on empirical data, is used in the finite element analysis of concrete specimens containing polymer self-healing microcapsules to quantify self-healing efficiency and local damage-healing behavior. The predicted increase in the self-healing rate is more pronounced for specimens subjected to compression compared to that for elements subjected to four-point bending. Thus, for a 20% increase in healing efficiency, strength recovery in compression increases from 18.5% to 32% for C25 and C30, respectively, whereas the corresponding values for tension in the tension-be-flexure setup are 3.5% and 5.3%.

## 1. Introduction

In concrete and, more generally, in cementitious materials, self-healing is conventionally associated with natural, or autogenous, processes resulting in crack sealing [1,2]. Cracked concrete exhibits a natural potential for self-healing. According to [3], calcium carbonate $\left(CaCO_{3}\right)$ precipitation plays a major role in the autogenous self-healing. Other mechanisms that reportedly contribute less include the continued hydration of unhydrated cement [4,5]; the swelling of the cementitious matrix [3]; and the crack blocking by suspensions or loose concrete particles [3].

To enhance autogenous healing, techniques employing crystalline admixtures (CAs), adhesive agents, and bacteria have become an active field of research. While CAs are usually incorporated directly into the matrix, the adhesive agent and bacteria require immobilization or encapsulation to avoid premature triggering. For this purpose, technologies employing micro- or macro-capsules or hollow “vascular” systems are being developed. A state-of-the-art review can be found in [6].

In a pioneering work by [7], the concept for self-healing is realized for polymer composites. To this end, microencapsulated healing agent and a catalytic chemical trigger are dispersed in the epoxy matrix. A propagating crack is supposed to destroy the shells of the microcapsules. The released healing agent reacts with the catalyst, and the formed polymer seals, fully or partially, the propagating crack.

The self-healing agent release described above is typically referred to as “mechanical triggering.” It imposes some requirements on the microcapsule shell, which should withstand mechanical actions during material preparation; at the same time, excessive rigidity would hinder its breakage. In addition, for cementitious composites and concrete, the application of microencapsulated self-healing agents is accompanied by issues, such as loss of fluidity [8], reduction in workability and strength [9], negative impact on rheology, and hydration exotherm (of the fresh mixture). The hydrophilicity of certain types of microcapsules [10,11] can lead to a porous (i.e., weak) interface between them and the cementitious matrix.

Researchers concentrate their efforts to mitigate these shortcomings by improving the shell design or chneanging the triggering mechanism. Some recent works study the modification of epoxy resin-ethyl cellulose microcapsules with nano-SiO_2_ to improve hydrophobicity [12], the coating of fly ash cenospheres with a nano-sized glass crystal film to enhance stiffness and hardness [13], nano-silica reinforcement of calcium alginate/epoxy resin capsule prepared at room temperature, or by using a green technology to achieve a strong bond with cementitious matrix [14]. Stimuli, independent of crack propagation, that reportedly ensure timely and more efficient triggering are ultrasonic waves [15,16], electromagnetic waves [17], and microwaves [18].

A novel method to produce high-strength capsules compatible with the cementitious matrix is proposed by [19]. It is noteworthy that these capsules fall beyond the definition of microcapsules. The range of technologies, the feasibility of which has been investigated for self-healing, includes 3D printing to produce macrocapsules (to provide a larger quantity of healing agent) [20,21,22,23] and vascularities [24]. Among the more “exotic” solutions to boost self-healing is the enzymatic one [25]. By carbonic anhydrase, the reaction between Ca^2+^ ions and atmospheric CO_2_ is catalyzed to form calcium carbonate crystals with thermomechanical properties similar to the cementitious matrix.

Despite significant research efforts to experimentally characterize the self-healing agents, quantify self-healing efficiency, and propose solutions to the shortcomings of self-healing technologies, their large-scale application necessitates adequate predictive models. The need for self-healing arises in the context of damage accumulation, nucleation of microcracks and microcavities, initiation and propagation of macrocracks. Based on these considerations, continuum damage mechanics (CDM) is deemed an adequate theory that describes the material response, including the phases prior to macro-crack formation.

Early contributions are those of Kachanov [26] and Rabotnov [27]. Specifically, Kachanov related material degradation to damage density, defined as the reduction in load-carrying area, resulting from damage accumulation on the microscopic scale. The scope of the article is confined to models formulated within the representative volume element (RVE). A damage variable can be formulated for an arbitrary plane in the material defined by its normal; physically, it quantifies the ratio of the nominal area of microcracks and cavities intersected by the plane to the initial area of the plane in the undamaged material. In accordance with the equivalence principle, the strain behavior of the damaged material can be obtained using the constitutive equation for the undamaged material and the effective stress (i.e., the stress modified by the damage variable) [28]. Generally, the damage is not isotropic and varies with the material directions. Therefore, the damage variable should be a tensor: a second-order tensor [29] or a fourth-order tensor [30]. Assuming the simplifying hypothesis for isotropic damage, the damage variable can be represented by a scalar.

To describe the emerging self-healing technologies, theoretical models rooted in CDM were extended to account for the reversibility phenomena resulting from the healing and gave rise to continuum damage-healing mechanics (CDHM). Mergheim and Steinmann [31] proposed a constitutive relationship to model damage, healing, and re-damage in already-healed material by using two distinct scalar damage variables. One accounts for damage accumulation in material not yet subject to healing, whereas the other evaluates the re-damaging process of the already healed material. Additionally, another scalar variable was formulated to account for healing. Barbero et al. [32] went beyond the isotropy. The authors considered anisotropic damage and healing in self-healing composite materials by defining rank-two damage and healing tensorial variables. It should be noted that the theory they outlined is based on the assumption that the growth directions of microcracks coincide with principal material directions in the composite material. Voyiadjis et al. [33] employed second-rank anisotropic damage and healing variable tensors and fourth-rank anisotropic damage and healing variable tensors to capture the irregular inelastic deformation of shape memory polymers (SMP) and polyethylene terephthalate (PET), as well as plastic and damage response of glassy polymers. The damage variable, in its tensorial form, quantifies the overall material degradation, including kinematic hardening (the latter represents the shift in the center of the damage surface), and isotropic hardening, which is related to the change in the size of the damage surface [34,35]. Abu Al-Rub et al. [36] extended Kachanov’s concept of effective undamaged configuration to enhance the capabilities for modeling micro-damage healing phenomena. Pan et al. [37] proposed a model for material containing distributed micro-capsules. The damage and healing behavior was considered in the context of the effective states. A healing function was formulated within the study, assumed to depend on the damage variable and the concentration of microcapsules. The authors applied the model to investigate the response of the self-healing material by considering a set of theoretical problems encompassing cylindrical specimens, plates, and beams.

Research on the damage variable as a mathematical and mechanical object continues. New formulations for the damage variable: logarithmic, exponential, etc., as well as definitions for damageability and integrity were proposed by [38]. A procedure to decompose the damage variable to decouple crack-induced damage from void-induced damage was proposed by [39].

Models rooted in CDM and CDHM generally have a strong thermodynamic basis. The works of Coleman and Gurtin [40] and Lubliner [41] have been extended to capture the behavior of materials with healing capabilities. Using the Clausius-Duhem inequality and the Helmholtz free energy function, Voyiadjis et al. [42] proposed a coupled elastoplastic-damage-healing formulation for an isothermal process, under the assumption of quasi-static loading and small displacements. The authors considered a thermodynamically consistent set of coupled constitutive equations accounting for plasticity, damage, and healing.

Coupled temperature-dependent viscoelastic, viscoplastic, visco-damage, and micro-damage constitutive equations that account for self-healing, rooted in CDM, were proposed by [43] and [44] with subsequent finite-element implementation to predict the mechanical response of asphalt concrete [45]. Finite-element analysis employing a cohesive-zone self-healing model was presented by [46].

A more detailed finite-element model, developed to study the interface between the capsule and the cementitious matrix during hydration and drying of the cement, was recently proposed by [47]. In line with the development of more detailed models, an approach employing finite-element modeling was used to simulate the diffusion and reaction kinetics of self-healing bacterial agents in concrete microstructures [48].

The study reported in this article employs an original model rooted in continuum damage mechanics designed to reproduce the response of concrete containing distributed microcapsules: a damageable material with a self-healing potential. The constitutive relationship is obtained by coupling linear elasticity with isotropic damage. The hypothesis is formulated that the isotropic model can reproduce the damage-healing response with sufficient accuracy. Based on theoretical considerations, the scalar damage variable does not capture effects such as damage-induced anisotropy. On the other hand, in a more general context and based on comparison against experimental data, this approach has proven well-suited to accurately reproduce the response of reinforced concrete structural elements [49]. A single variable is used herein to account for both damage and healing. The algorithmic approach accounts for damage, healing, and further damage accumulation in the healed material. The damage distribution and, respectively, material properties degradation taking place with damage accumulation as well as their redevelopment with healing are tracked throughout the loading history. By assumption, the healing is triggered at a user-specified appropriate moment. The response of standard cylindrical specimens loaded in compression and prismatic specimens loaded in tension by flexure is simulated to shed light on phenomena associated with damage and healing. The current study builds upon that presented in [50].

The manuscript is organized as follows. First, the employed material model and numerical algorithm are outlined in Section 1 and Section 2 entitled “The material model,” and “The numerical algorithm,” respectively. A common testing protocol is assumed for all numerical experiments. It is outlined in Section 3. In a pre-loading phase, specimens are loaded until a specified damage distribution is obtained. After that, specimens are unloaded and subjected to healing. In the healing phase, the polymerized product of the healing agent seals, either entirely or partially, the microcracks and microcavities formed in the pre-loading phase. In the employed algorithm, the healing efficiency depends on the amount of the healing agent and the damage levels reached in the pre-loading phase. Therefore, a certain damage level may still be present after the healing is completed. In the post-healing phase, specimens are loaded again until they collapse.

Numerical results obtained using the above-mentioned testing protocol are presented and discussed. Two setups: compression and tension by flexure are considered in Section 4 and Section 5. For each setup, the geometry and boundary conditions are described in detail. Numerical simulations are performed for three concrete grades: C25, C30, and C35. The characteristics of the undamaged material (elasticity modulus and Poisson’s ratio) corresponding to each concrete grade are set, as defined in the standard guidelines (for example, [51]), and the model constants are identified via curve fitting targeting the expected value of the maximum stress. Additionally, for each concrete grade, the amount of encapsulated healing agent is varied (introduced through the healing efficiency parameter, defined later). After that, the testing protocol comprising the pre-loading-, healing-, and secondary loading- (or post-healing) phases is run. Based on the numerical results obtained by simulating the global response of specimens containing a self-healing agent, the regain in load-carrying capacity is predicted. The local response is studied by monitoring the damage variable evolution throughout the loading history in selected finite elements.

## 2. Materials and Methods

The presented investigation mainly relies on a constitutive relationship, rooted in continuum damage mechanics. The chosen material model is implemented by creating a script for a general-purpose finite element code (ANSYS 19.0 Mechanical APDL). Furthermore, a testing protocol is developed to perform numerical experiments designed to quantify the efficiency of the self-healing. Both the constitutive relationship for concrete and the protocol developed for the numerical testing are outlined below.

### 2.1. The Material Model

The nonlinear response of concrete is modeled by coupling linear elasticity with isotropic damage [28,52] as follows:

(1) $$\bar{\bar{\sigma }}=\frac{\nu }{\left(1+\nu \right)\left(1-2\nu \right)}E_{0}\left(1-D\right)tr\left(\bar{\bar{\varepsilon }}\right)\bar{\bar{I}}+\frac{1}{\left(1+\nu \right)}E_{0}\left(1-D\right)\bar{\bar{\varepsilon }},$$

where $\overset{̿}{\sigma }$ is the stress tensor, $E_{0}$ is the elasticity modulus of the undamaged material, D denotes the damage variable, $\overset{̿}{\varepsilon }$ is the strain tensor, $tr\left(\overset{̿}{\varepsilon }\right)$ is the trace of the strain tensor, $\overset{̿}{I}$ is the unit tensor, and ν is the Poisson’s ratio. The model presumes that damage evolution does not affect the material symmetries. The rate of change of the damage variable depends on a set of model constants and a variable $\varepsilon_{eqv}$ referred to as ‘equivalent strain’ [53,54],

(2) $$\varepsilon_{eqv}=\sqrt{\sum_{j=1}^{3}{\langle \varepsilon_{j}\rangle }^{2}},j=1\ldots 3,\langle \varepsilon_{j}\rangle =\frac{1}{2}\left(\varepsilon_{j}+\left|\varepsilon_{j}\right|\right).$$

In Equation (2), $\varepsilon_{j}$ are principal strains. Damage accumulates, provided the equivalent strain is greater than the damage threshold $\varepsilon_{0}$,

(3) $$D=D^{\left(c\right)}+\beta D^{\left(t\right)},$$

(4) $$\begin{array}{l} D^{\left(c\right)}=1-\varepsilon_{0}\left(1-A_{c}\right)/\varepsilon_{eqv}-A_{c}/\exp\left(B_{c}\left(\varepsilon_{eqv}-\varepsilon_{0}\right)\right),\\ D^{\left(t\right)}=1-\varepsilon_{0}\left(1-A_{t}\right)/\varepsilon_{eqv}-A_{t}/\exp\left(B_{t}\left(\varepsilon_{eqv}-\varepsilon_{0}\right)\right), \end{array}$$

(5) $$\begin{array}{c} \begin{array}{ccc} \frac{dD}{dt}=0 & if & \varepsilon_{eqv}<\varepsilon_{0} \end{array}\\ \begin{array}{ccc} \frac{dD}{dt}>0 & if & \varepsilon_{eqv}\geq \varepsilon_{0} \end{array} \end{array}.$$

In Equation (3), $D^{\left(c\right)}$ and $D^{\left(t\right)}$ denote damage variable components for compression and tension, respectively, and β is a model constant; in Equation (4), $A_{c}$, $B_{c}$, $A_{t}$, and $B_{t}$ are model constants; the damage threshold, $\varepsilon_{0}$, is another model constant that should be identified based on experimental data.

Local failure occurs when the critical value of the damage variable, $D_{c}$, is reached; $D_{c}$ should also be experimentally identified. Local failure denotes the collapse of the representative volume element [55]. Based on an analogy between the ‘representative volume element’ defined in continuum damage mechanics and the ‘finite element’ employed in the finite-element analyses, the constitutive relationship, Equation (1), can be implemented in finite element simulations.

### 2.2. The Numerical Algorithm

**Figure 1:**
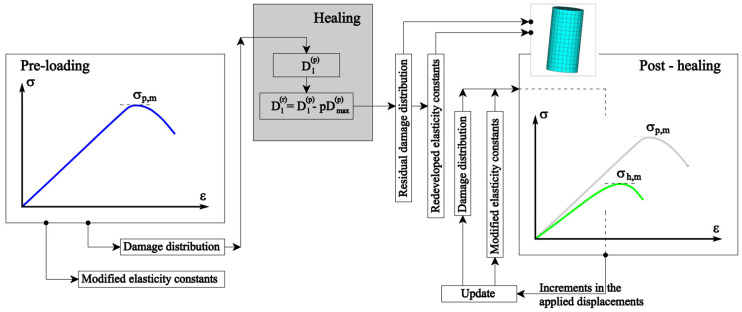
A flow-chart of the numerical algorithm employed to simulate the self-healing triggered after the pre-loading phase (blue) and before the post-healing phase (green).

All simulations presented herein include three phases: a preloading phase, a healing phase, and a post-healing phase, strictly following this sequence. An original numerical procedure quantifies the mechanical damage accumulated in the material and modifies its mechanical properties accordingly to simulate a strain-softening response, as defined in Equations (1)–(5). Self-healing is incorporated in this framework again through the damage variable. The nucleation and growth of microcracks in the material during the pre-loading phase are reflected in the increase of the damage variable. When self-healing is triggered, the healing agent seals the microcracks and, as a result, the net resisting area increases. The mechanical characteristics of the material are restored, and the damage variable decreases. The numerical algorithm is schematized in Figure 1.

#### 2.2.1. The Pre-Loading Phase

Within the pre-loading phase, specimens are loaded to generate damage distribution. The goal is to create prerequisites for self-healing, without reaching global failure. It has been considered appropriate to avoid macroscopic crack initiation in this phase. A stress-based criterion can be used to limit the pre-loading phase: the material can be loaded to some fraction of the maximum allowable stress. In contrast, a damage-based criterion is preferred herein: i.e., the quasistatic load is increased until a predefined level of damage is reached. This damage level is defined as a portion of the critical value of the damage variable. At the end of the pre-loading phase, the damage distribution in the considered specimens is determined elementwise, along with the accordingly modified material constants.

#### 2.2.2. The Healing Phase

After pre-loading, specimens are completely unloaded, and the self-healing is triggered. The released healing agent seals the formed micro-cracks and micro-cavities. The cross-sectional area resisting the applied load is increased. In the model, this is accounted for by a drop in the damage variable in zones where the material is healed.

It is assumed in the model that healing depends on a parameter p that takes into account the amount and characteristics of the healing agent. This parameter can be considered an indicator of the extent to which micro-cracks and micro-cavities formed in the pre-loading phase can be sealed with the available healing agent. As shown in Figure 1, within the healing phase, the damage variable evaluated for each element is compared with the product $pD_{max}^{\left(p\right)}$, $D_{max}^{\left(p\right)}$ being the prescribed maximum value of the damage variable at the end of the pre-loading phase, and p—the healing efficiency parameter. The residual damage distribution, after the healing phase, is defined as follows:

(6) $$D_{l}^{\left(r\right)}=D_{l}^{\left(p\right)}-pD_{\max}^{\left(p\right)},\;D_{\max}^{\left(p\right)}=\max\left\{D_{l}^{\left(p\right)}\right\}\;D_{l}^{\left(r\right)}\geq 0\;l=\left\{1\ldots \left.N\right\}\right.,$$

In Equation (6), $D_{l}^{\left(r\right)}$ is the residual damage, which quantifies the amount of unhealed microcracks after the healing phase in the finite element number l, $D_{l}^{\left(p\right)}$ is the damage distribution defined element-wise after the pre-loading phase, and N is the total number of finite elements in the considered finite-element model.

#### 2.2.3. The Post-Healing Phase

After the self-healing process is triggered and completed, the damage variable is set back to zero in finite elements that can be completely healed, according to the criterion formulated in Equation (6), and decreased in finite elements that allow for partial healing, according to the same criterion. Healed specimens are then loaded again until failure.

The self-healing efficiency is evaluated based on the results obtained in the post-healing phase. The maximum load-carrying capacity reached in the post-healing phase, in terms of maximum stress or maximum applied load, is compared with the maximum stress or maximum load obtained for a pristine specimen.

**Figure 2:**
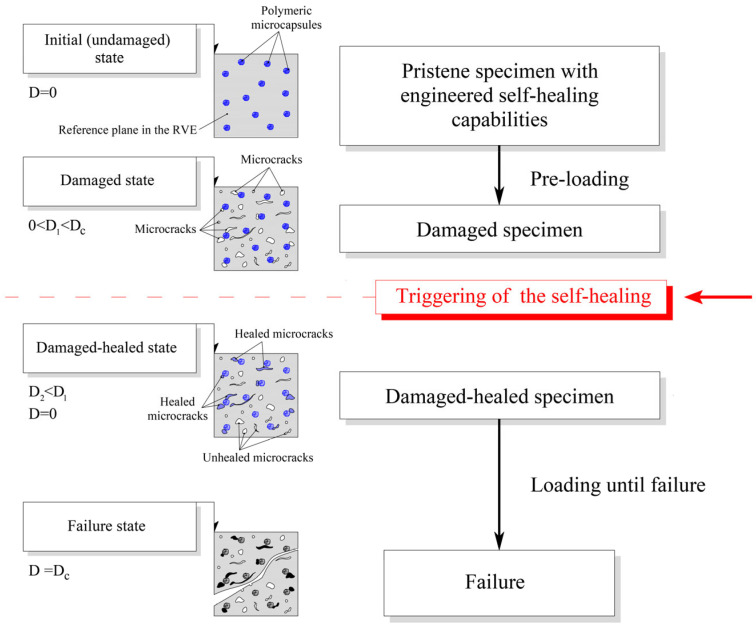
Testing protocol realized in numerical simulations along with hypothetical visualization of damage and healing phenomena in an arbitrary plane within the RVE. The activation of self-healing (red arrow) stops the mechanical degradation. After this external intervention, the material properties begin to recover.

The idealized damage and healing response of concrete specimens containing polymeric self-healing microcapsules is illustrated in Figure 2.

## 3. Results

Results obtained by finite element analysis are presented in this section. Specifically, the damage-healing behavior of plain concrete cylindrical specimens loaded in compression and plain concrete prismatic specimens subjected to tension by flexure is investigated. Both global and local results are discussed, the former in terms of stress–strain relationships that reveal the material response, and the latter in terms of damage distribution, as well as its evolution. Based on the global stress–strain relationships obtained for the pristine specimen and after healing, a quantitative criterion is implemented to evaluate the self-healing efficiency.

### 3.1. Response of Specimens Loaded in Compression

#### 3.1.1. Geometry and Boundary Conditions

**Figure 3:**
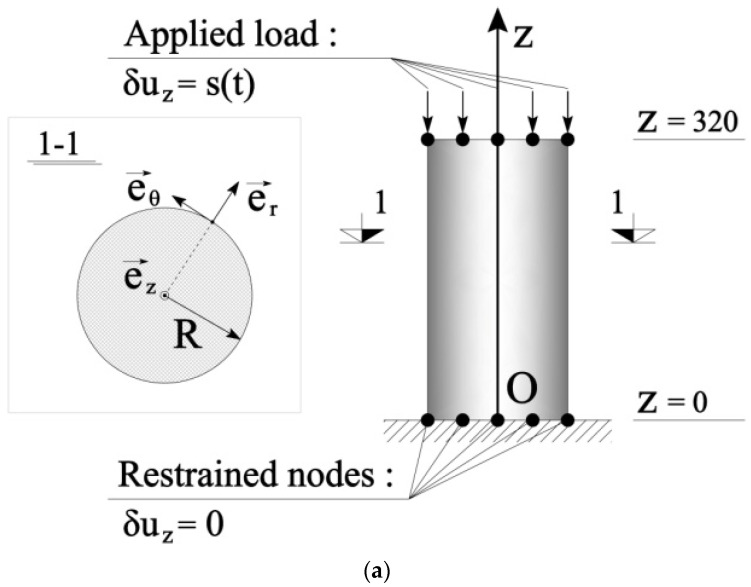

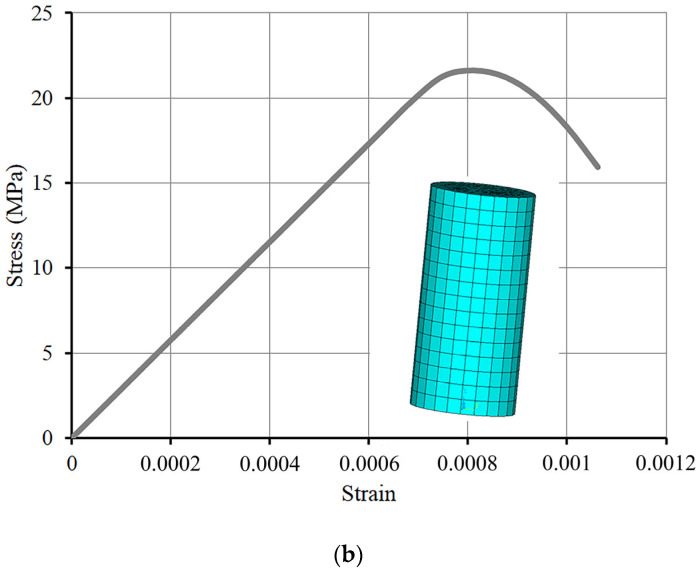
Schematization of the boundary conditions and the testing protocol (**a**); visualization of the generated mesh (set 1 in Table 1) and stress–strain relationship obtained by finite element analysis (**b**).

Numerical simulations include displacement-controlled characterization tests on standard cylindrical concrete specimens with a circular cross-section $\left(R=80\;\mathrm{mm}\right),$ and 320 mm in height (Figure 3a). Displacements of nodes generated at the bottom surface $\left(z=0\right)$ in the finite element model are restrained, whereas vertical displacements are incrementally applied to nodes at the top surface, initially situated at z=320, as shown in Figure 3a. The employed boundary and initial conditions are provided below, using a cylindrical coordinate system $\left(0,\;r,\;\theta ,\;z\right)$:

(7) $$\begin{array}{c} u_{z}(r,0,t)=0,\sigma_{zr}(r,0,t)=0,\;\mathrm{for}\;0\leq r\leq R,\\ u_{z}(r,320,t)=-D(t),\sigma_{zr}(r,320,t)=0,\;\mathrm{for}\;0\leq r\leq R,\\ \sigma_{rr}(R,z,t)=0,\sigma_{rz}(R,z,t)=0.\;\mathrm{for}\;0\leq z\leq 320, \end{array}$$

(8) $$u_{i}\left(r,z,0\right)=0,\frac{\partial u_{i}}{\partial t}\left(r,z,0\right)=0,i=\left\{r,\left.z\right\}\right.,0\leq r\leq R,0\leq z\leq 320.$$

In Equations (7) and (8), $u=\left\{u_{r},u_{\theta },u_{z}\right\}$ and $\overset{̿}{\sigma }=\left\{\sigma_{rr},\;\sigma_{\theta \theta },\sigma_{zz},\sigma_{\theta z},\sigma_{rz},\sigma_{r\theta }\right\}$ are the displacement vector and the stress tensor, respectively; $D\left(t\right)=\Delta s\cdot t$ denotes the incremental displacement applied to the specimen’s top surface, Δs=Const is the displacement increment, and t is the time. Despite the problem being stated as time-dependent in Equations (7) and (8), only cases of quasi-static loading are considered in the performed analyses, i.e., Δs is sufficiently small.

For the mesh generation, SOLID185 finite element is employed. SOLID185 has eight nodes with three degrees of freedom per node, translations along the axes of the nodal coordinate system. A tentative finite-element mesh is depicted in Figure 3b.

The stress–strain relationship in the pre-loading phase is simulated for four sizes of the generated finite-element mesh (Table 1) to investigate possible mesh dependency in the performed analyses. All four simulations performed for the four generated finite-element meshes showed practically identical responses. The four stress–strain curves merge into one, depicted in Figure 3b.

Results obtained using the finite element mesh labeled ‘Set 1’ are taken as reference and their similarity with results obtained with the other meshes is evaluated. The similarity index is calculated according to [56],

(9) $$\begin{array}{c} I_{s}=1-\frac{\sqrt{{\left(V_{i}^{\left(n\right)}-V_{i}^{\left(R\right)}\right)}^{2}}}{\sqrt{{\left(V_{i}^{\left(R\right)}-V_{m}^{\left(R\right)}\right)}^{2}}},i=1\ldots n,\\ V_{m}^{\left(R\right)}=\sum_{i=1}^{n}V_{i}^{\left(R\right)}/n. \end{array}$$

Equation (9) compares two datasets, one containing the stresses in the reference curve and the other, the stresses in the curve to be assessed for similarity. In Equation (9), $V_{i}^{\left(R\right)}$ and $V_{i}^{\left(n\right)}$ are the stresses at a given strain level in the reference dataset and the dataset to be compared with the reference one, respectively; $V_{m}^{\left(R\right)}$ is the mean of the reference dataset, and n is the number of elements in each dataset. A higher value of the similarity index corresponds to a better fit between the compared datasets; a similarity index of 1 would denote a perfect fit. The good fit between the results obtained for various characteristics, in terms of maximum element size, of the generated mesh demonstrates that, in the pre-loading phase, the numerical solution is not mesh-sensitive.

**Figure 4:**
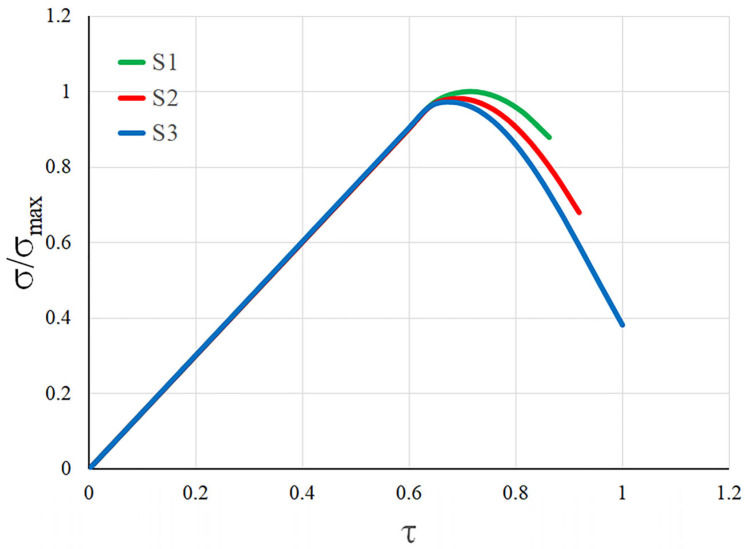
Sensitivity study on time-step size; results obtained for simulations S1, S2, and S3 are plotted in green, red, and blue, respectively.

Additionally, a possible dependency on the rate of change of the applied displacement in the simulation of the quasi-static response was investigated (Figure 4).

In Figure 4, normalized stress is plotted against normalized time, *τ*. The total displacement is realized in 23 steps for S1, 27 steps for S2, and 31 steps for S3. Within each step, the minimum, the maximum, and the recommended number of sub-steps are 100, 150, and 120, respectively. Time is normalized with respect to the time (in the numerical experiment) needed to accomplish the slowest simulation (S3). Stresses are normalized with respect to the maximum stress achieved in the fastest simulation (S1). The ratio of the maximum achieved stresses in the simulations is 1:0.98:0.97. This result, together with the outcomes of the finite element size sensitivity study mentioned above, demonstrates the robustness of the algorithm.

#### 3.1.2. Model Constants Identification

**Figure 5:**
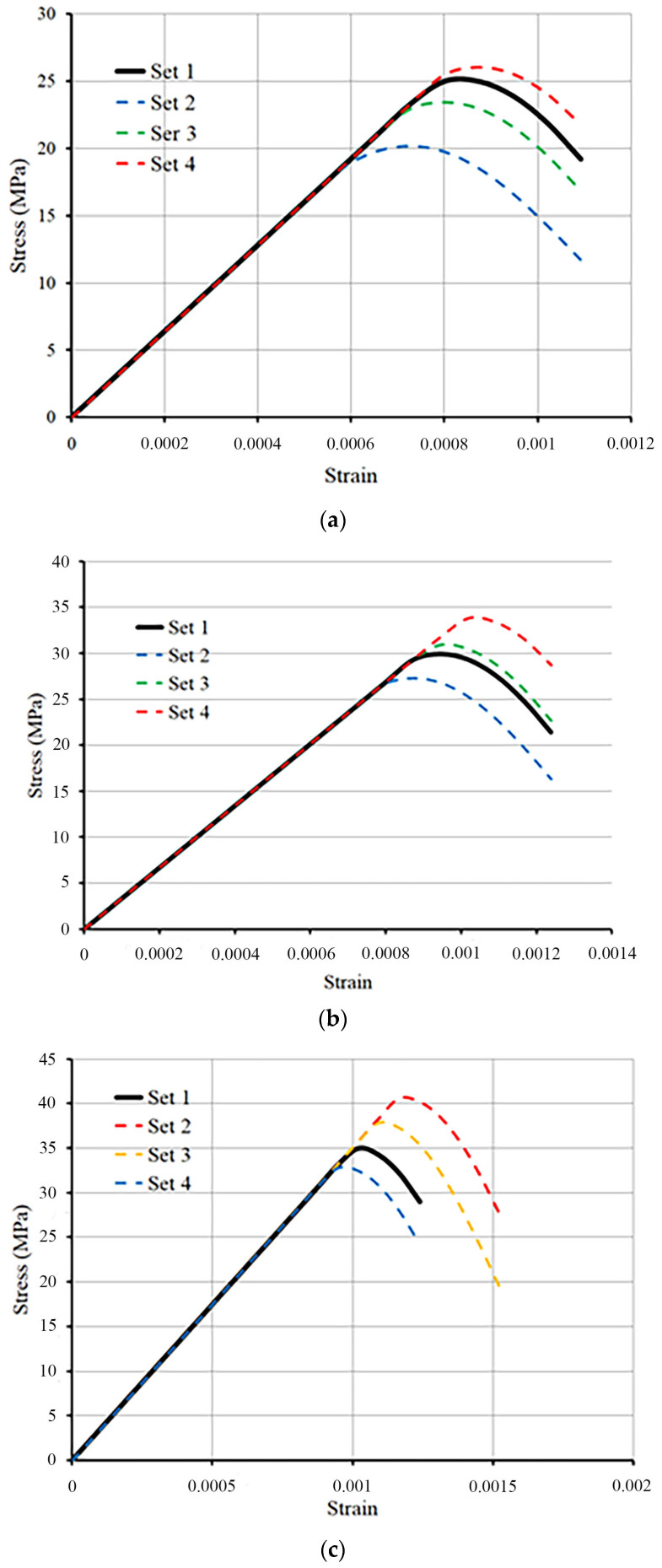
Model constant identification for various concrete grades: C25 (**a**); C30 (**b**); and C35 (**c**); the best fit is plotted in a black continuous line.

Numerical experiments start with the identification of the model constants. Figure 5 depicts the final stages of the identification process for concrete grades C25, C30, and C35. The identified model constants are subsequently employed in the pre-loading and the post-healing phases.

The input data include the Young’s modulus of the undamaged material, and the best set of model constants yields the targeted material strength (e.g., the compressive strength). Both Young’s modulus and compressive strength are known for a given concrete grade. In such cases, a searching algorithm can accelerate convergence. A genetic algorithm using value-based encoding, stochastic universal sampling, and a single-point crossover was employed. For example, to identify the model constants that govern the compressive response, a chromosome containing three substrings {*ε*_0_ *A_t_* *B_t_*} was defined.

#### 3.1.3. Simulation of the Self-Healing

- Global response

**Figure 6:**
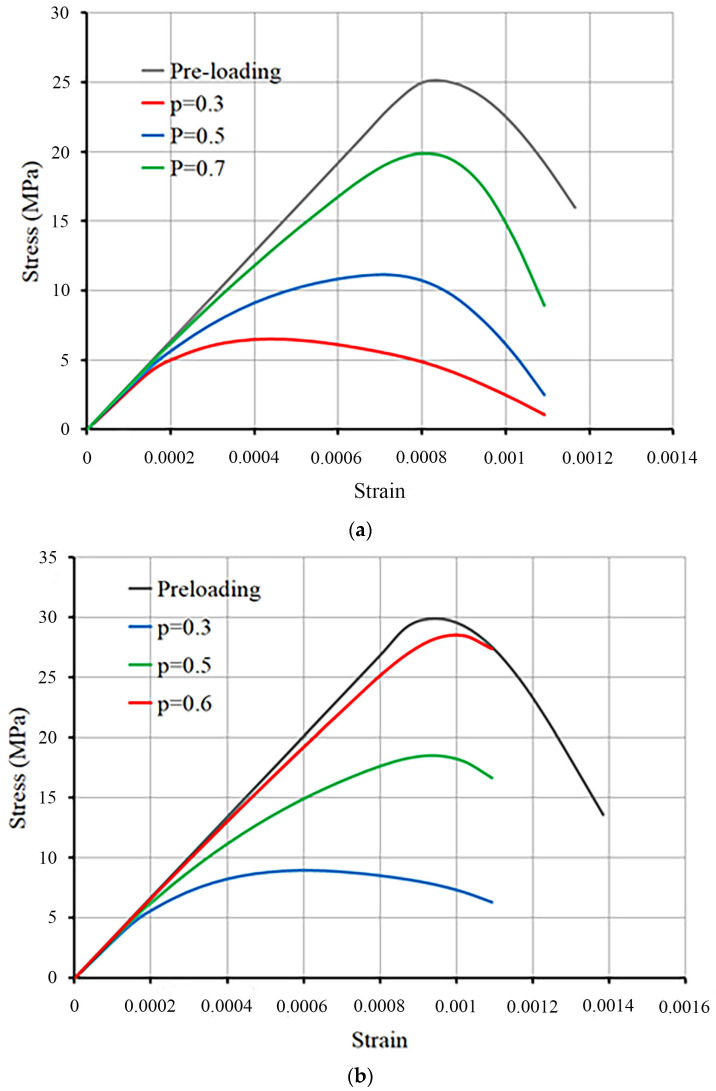

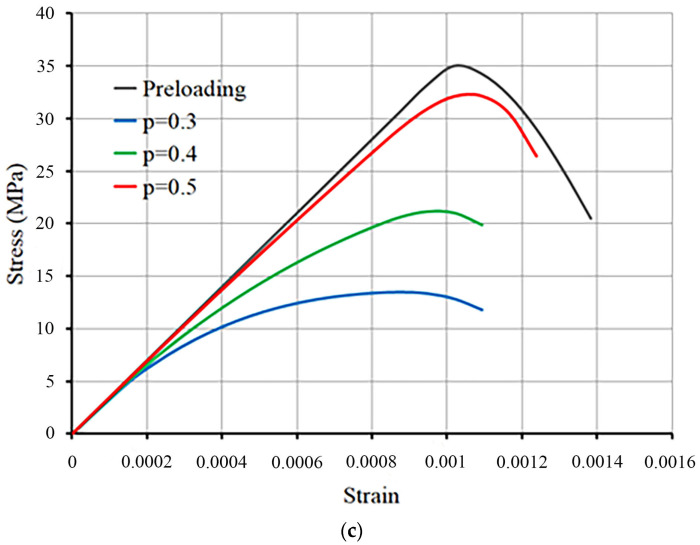
The global response of the specimens in the pre-loading phase (in grey) and in the post-healing phase, in the function of the parameter p (in red, green, and blue) for: C25 (**a**); C30 (**b**), and C35 (**c**).

Figure 6 presents the behavior of the healed material compared with its response in the pre-loading phase, all expressed through the numerically obtained stress–strain relationships for concrete grades C25, C30, and C35. Various responses in the post-healing phase are obtained by varying the healing efficiency parameter p.

Following [57], an index of strength recovery is evaluated. Using the notations introduced in Figure 1,

(10) $$I_{R}^{\left(c\right)}=\frac{\sigma_{h,m}-\sigma_{p,p}}{\sigma_{p,m}-\sigma_{p,p}}.$$

In Equation (10), $\sigma_{h,m}$ denotes the maximum compression stress reached in the post-healing phase, $\sigma_{p,p}$ is the stress at the end of the unloading that follows the preloading phase, and $\sigma_{p,m}$ is the maximum compression stress for the considered material. Within the employed simulation protocol, specimens are completely unloaded after the pre-loading phase, which yields $\sigma_{p,p}=0$. The estimates for the strength recovery in compression are summarized in Table 2.

Specimens of all series (C25, C30, and C35) are pre-loaded to generate approximately identical damage levels. Maximum damage after the pre-loading phase is $D_{max}^{\left(p\right)}=0.38$ for C25, $D_{max}^{\left(p\right)}=0.41$ for C30, and $D_{max}^{\left(p\right)}=0.42$ for C35. Analyzing the results summarized in Table 2, it is apparent that the compressive strength recovery is more pronounced with the increase in the concrete grade. For example, for p=0.5, the estimated strength recovery indices are $I_{R}^{\left(c\right)}=44.6\%$, $I_{R}^{\left(c\right)}=62\%$, and $I_{R}^{\left(c\right)}=91.7\%$, for C25, C30, and C35, respectively. The same tendency is observed for the other healing efficiency parameters employed.

For engineering purposes, the model can be tuned to match the response obtained in experiments, for example, those reported in [57], by accounting for the employed experimental setup. Thus, the parameter p is reconstructed (identified) from the empirically observed macroscopic behavior of specimens in which healing is triggered after pre-damage (Table 3).

Result #6 is apparently inconsistent with the applied technology, which does not produce superhealing effects (i.e., healing ratios greater than 100%). The reconstruction error, $\frac{\left|x_{i}-x_{t}\right|}{x_{1}}\times 100$, for each result $x_{i}$, is calculated with respect to the experimentally obtained target value, $x_{t}=87.8\%$. A smaller value of the reconstruction error indicates a better fit. Although it provides a slightly conservative prediction, the best candidate among those listed in Table 3 is #5, i.e., p=0.22.

It should be noted that the constitutive relationship for the self-healing material reported in the article operates on the mesoscopic scale, i.e., within the RVE. A more refined model, which represents the microcapsules with their shells, the shells’ breakage under the current stress state, the spreading of the healing agent due to capillary action, and the subsequent polymerization of the healing agent, can provide a basis to quantify the healing variable. This model could be employed as an alternative perspective on the experimental results, instead of using the experimental data to calibrate the material model formulated at the mesoscopic scale. The development of such a model will be the subject of forthcoming research. In [7], similar modeling was performed with the Eshelby-Mura equivalent inclusion method [58]. More recently, similar problems have been discussed in [59].

- Local response

**Figure 7:**
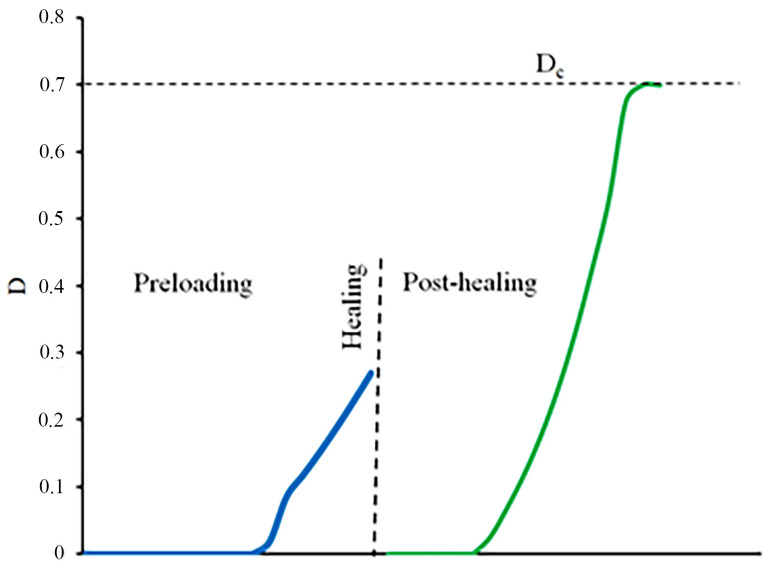
Damage evolution in an arbitrary finite element in the preloading and the post-healing phases.

Expressed through the damage variable evolution, calculated for one specified finite element, the local material response in the pre-loading phase, the healing phase, and the post-healing phase, is illustrated in Figure 7. In the preloading phase, damage in the considered finite element starts to accumulate as soon as the equivalent strain exceeds the lower damage threshold (the blue curve in Figure 7). For the assumed healing efficiency parameter, within the healing phase, the portion of the material corresponding to the considered finite element is completely healed, and the damage variable is set back to zero before the post-healing phase. In other words, in this case, the self-healing technology applied possesses the capacity to completely seal the micro-cracks or micro-voids generated in the preloading phase. On the contrary, if the amount of micro-cracks generated in the pre-loading phase requires a larger quantity of healing agent than initially provided in the material, a residual value of the damage variable will be returned after the healing phase $\left(D^{\left(r\right)}>0\right)$. For the finite element considered, curing process outcomes are accounted for by setting back the damage variable to zero. Within the analysis, the time needed for self-healing is neglected. The damage variable is reset at the moment in the loading history coinciding with the dotted line in Figure 7. Applying the quasi-static load in the post-healing phase provokes a secondary damage accumulation, expressed through the damage variable growth in the already healed element (the green line in Figure 7). The damage variable eventually reaches the critical value, $D_{c}$, and the finite element fails. Theoretically, local failure corresponds to a macro-crack initiation. Technically, such a finite element is deactivated, i.e., it does not contribute to the overall stiffness in the subsequent stages of the solution. This procedure is applied element-wise in the numerical solution.

### 3.2. Response of Specimens Loaded in Tension by Flexure

#### 3.2.1. Geometry and Boundary Conditions

**Figure 8:**
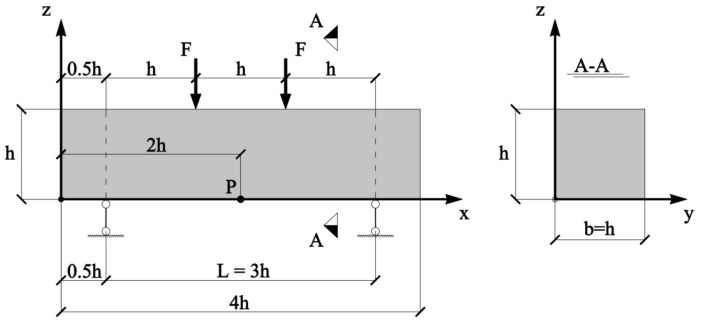
Scheme of the specimen for tension by flexure characterization setup.

All results in this subsection are obtained using the model depicted in Figure 8. The prismatic specimen with a square cross-section of height h and width $b\left(h=b\right)$ has a span L=3h. The distance from each load application point to the nearest support is h. The length of the specimen is 4h. The distance h is set equal to 1000 mm.

The following boundary conditions apply. On the bottom surface, z=0,

(11) $$\begin{array}{c} \sigma_{zz}(x,y,0,t)=0,\sigma_{zx}(x,y,0,t)=0,\cdot \sigma_{zy}(x,y,0,t)=0,\\ \;\mathrm{for}\;0\leq x\leq 4h,0\leq y\leq h, \end{array}$$

except for the lines where the vertical supports are modeled,

(12) $$u_{z}(0.5h,y,0,t)=0\;\mathrm{and}\;u_{z}(3.5h,y,0,t)=0,\;\mathrm{for}\;0\leq y\leq h,$$

On the top surface, z=h,

(13) $$\begin{array}{c} \sigma_{zz}(x,y,h,t)=0,\sigma_{zx}(x,y,h,t)=0,\sigma_{zy}(x,y,h,t)=0,\\ \;\mathrm{for}\;0\leq x\leq 4h,0\leq y\leq h, \end{array}$$

except for the lines where the load is incrementally applied,

(14) $$u_{z}(1.5h,y,h,t)=-D_{1}(t)\;\mathrm{and}\;u_{z}(2.5h,y,h,t)=-D_{1}(t),\;\mathrm{for}\;0\leq y\leq h$$

The lateral surfaces are not loaded:

(15) $$\begin{array}{c} \sigma_{xx}(0,y,z,t)=0,\;\sigma_{xy}(0,y,z,t)=0,\;\sigma_{xz}(0,y,z,t)=0,\\ \mathrm{for}\;0\leq y\leq h\;\mathrm{and}\;0\leq z\leq h,\\ \sigma_{xx}(4h,y,z,t)=0,\;\sigma_{xy}(4h,y,z,t)=0,\;\sigma_{xz}(4h,y,z,t)=0,\\ \mathrm{for}\;0\leq y\leq h\;\mathrm{and}\;0\leq z\leq h,\\ \sigma_{yy}(x,0,z,t)=0,\;\sigma_{yx}(x,0,z,t)=0,\;\sigma_{yz}(x,0,z,t)=0,\\ \mathrm{for}\;0\leq x\leq 4h\;\mathrm{and}\;0\leq z\leq h,\\ \sigma_{yy}(x,h,z,t)=0,\;\sigma_{yx}(x,h,z,t)=0,\;\sigma_{yz}(x,h,z,t)=0,\\ \mathrm{for}\;0\leq x\leq 4h\;\mathrm{and}\;0\leq z\leq h. \end{array}$$

The quasi-static load in Equation (14) reads $D_{1}\left(t\right)=\Delta s_{1}\cdot t$, where $\Delta s_{1}=Const$ denotes the applied displacement increment and t is the time.

#### 3.2.2. Model Constants Identification

**Figure 9:**
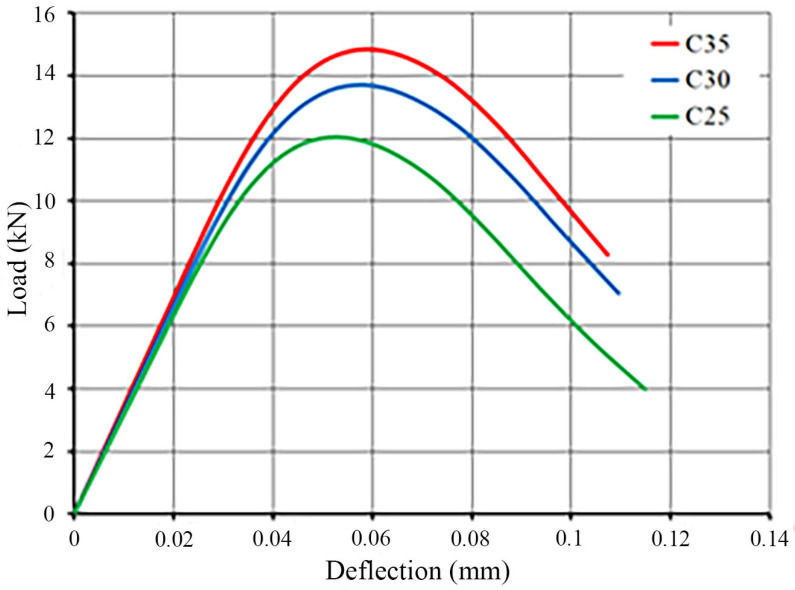
Load-deflection response of specimens subjected to tension by flexure if self-healing is not triggered.

The global response of specimens is obtained in terms of load-deflection response and stress evolution at the mid-span on the soffit surface (point $P\left(2h,\;0,0\right)$ in Figure 8). The behavior of concrete, grades C25, C30, and C35, is modeled and simulated. The numerically obtained load-deflection response is plotted in Figure 9.

**Figure 10:**
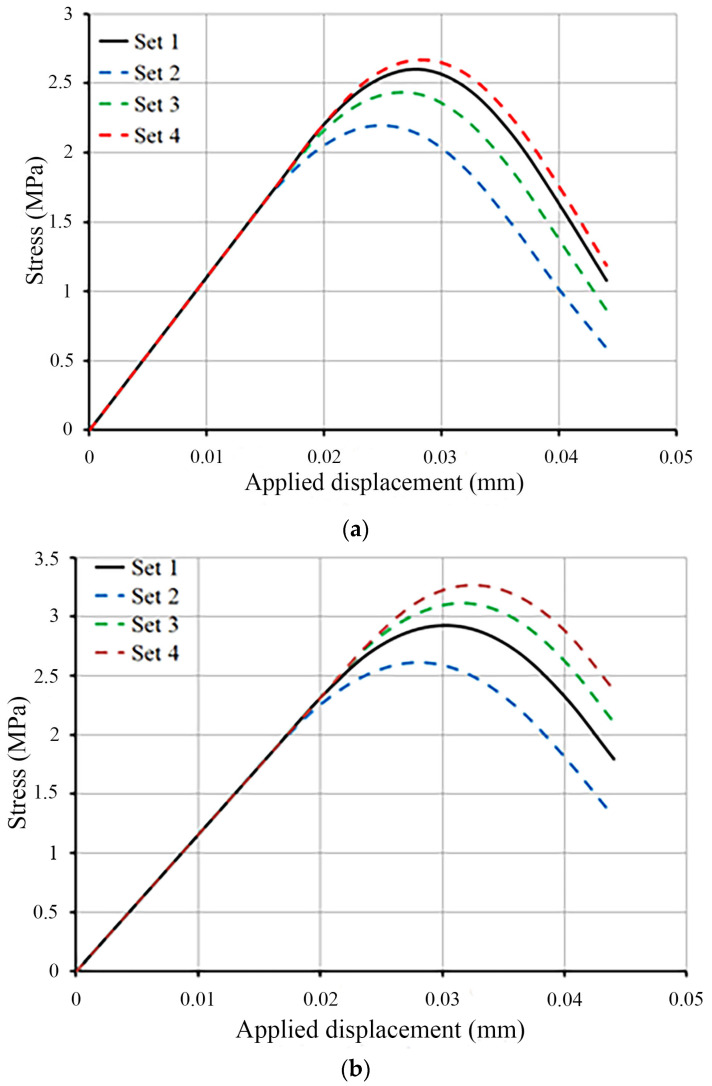

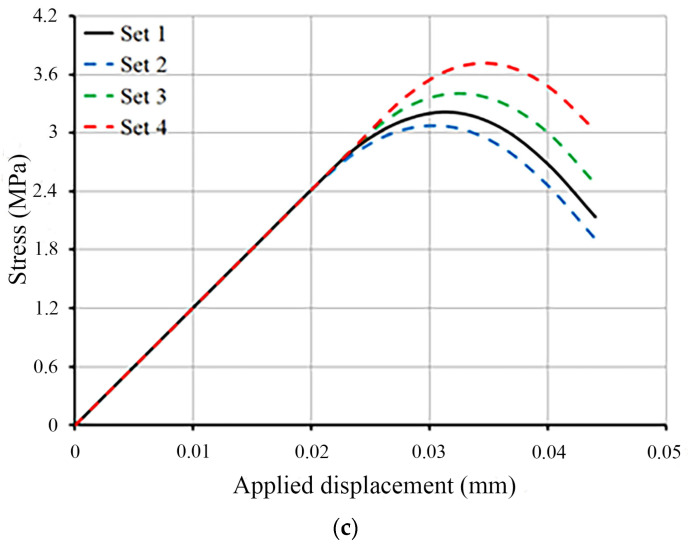
Model constants identification for concrete grades C25 (**a**), C30 (**b**), and C35 (**c**).

First, the model constants are identified, as shown in Figure 10a–c. To this end, the evolution of the maximum tensile stress is monitored. The elasticity modulus and the Poisson’s ratio of the undamaged material for the considered concrete grade are taken as an input, and model constants are varied to match the specified tensile strength: 2.6 MPa, 2.9 MPa, and 3.2 MPa for C25, C30, and C35, respectively.

#### 3.2.3. Simulation of the Self-Healing

- Global response

**Figure 11:**
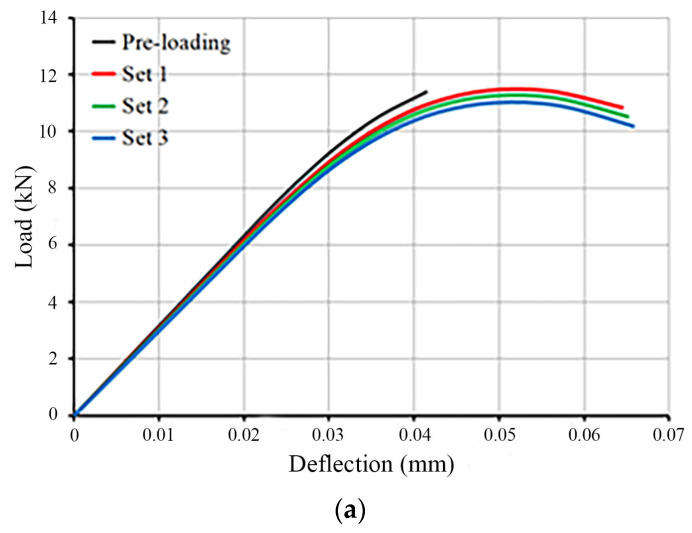

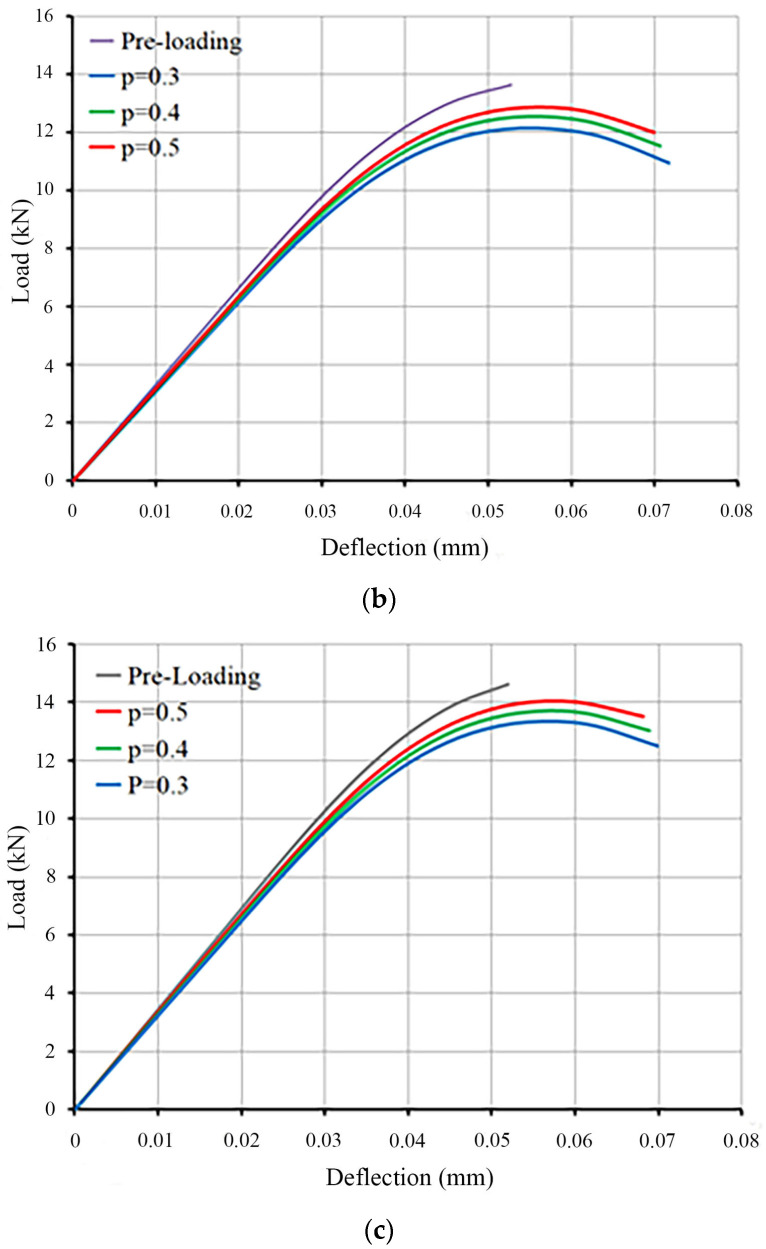
Response of prismatic concrete specimen subjected to four-point bending in the pre-loading phase (grey curve) and the post-healing phase for various values of the healing efficiency parameter p; results are provided for: C25 (**a**); C30 (**b**); and C35 (**c**).

After the model constants identification, simulations that outline the self-healing effects are performed. Specimens are loaded to generate an initial damage distribution. After that, they are completely unloaded, and the self-healing is triggered. When self-healing is complete, the specimens are loaded again until global failure is reached. For the tension by flexure numerical experiments, the response of all concrete grades is studied by assuming identical values of the healing efficiency parameter: p=0.3, 0.4, and 0.5. The obtained results are plotted in Figure 11a–c.

Equation (10) is slightly modified to evaluate the self-healing efficiency for specimens subjected to tension by flexure. In this case, the index of strength recovery is evaluated as the ratio of the maximum load reached in the post-healing phase $\left(F_{h,m}\right)$ to the maximum load that could be obtained for a pristine specimen without triggering the self-healing $\left(F_{p,m}\right)$, as follows:

(16) $$I_{R}^{\left(t\right)}=\frac{F_{h,m}}{F_{p,m}}.$$

The results illustrating partial strength recovery, depending on the concrete grades and the healing efficiency parameter, are summarized in Table 4.

The comparison (in Table 4) of the strength recovery indices $I_{R}^{\left(t\right)}$ corresponding to a given healing efficiency parameter p and various concrete grades shows that no clear trend can be formulated. On the contrary, for a given concrete grade, the increase in p always leads to a higher strength recovery ratio $\left(I_{R}^{\left(t\right)}\right)$. However, in the case of tension by flexure loading, the results for a specified concrete grade and various values of the healing efficiency parameter, $I_{R}^{\left(t\right)}$ diverge considerably less than those obtained for the specimens loaded in compression $\left(I_{R}^{\left(c\right)}\right)$. Thus, the increase in $I_{R}^{\left(t\right)}$, for an increase in p equal to 0.2, is 3.5%, 5.3%, and 4.6% for C25, C30, and C35, while for the same variation in p, the increase in $I_{R}^{\left(c\right)}$ is 18.5%, 32%, and 53.2% for C25, C30, and C35, respectively. A possible explanation for this observation might be related to the fact that the stress state in the cylindrical specimen loaded in compression is mostly uniaxial, while the prismatic specimen for the tension by flexure test is loaded in four-point bending. The stress state in the cylindrical specimen is supposed to be uniaxial compression, except for the narrow zones near the top and bottom faces. In contrast, the tension zone in the four-point bending specimen is below the neutral axis and between the supports. Also, the crack propagation in the prismatic specimen is fast, and the ‘window’ between the macro-crack initiation and failure is notably smaller compared to that in the cylindrical specimen loaded in compression.

- Local response

**Figure 12:**
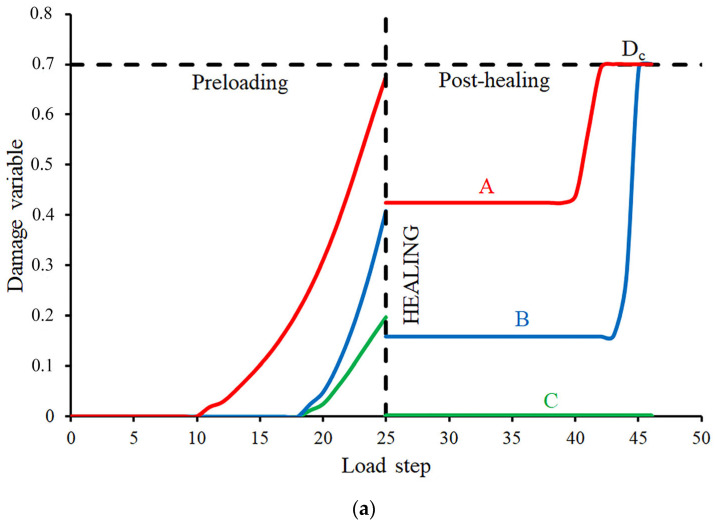

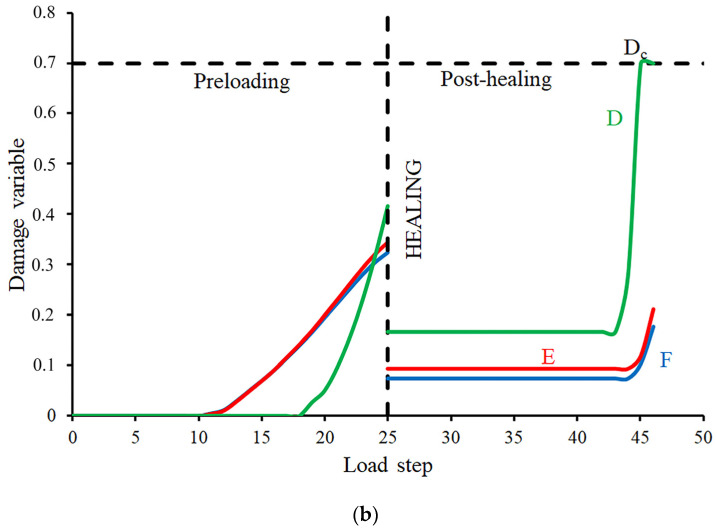
Local behavior: damage evolution in finite elements: A, B, and C (**a**); D, E, and F (**b**).

Figure 12 provides an insight into the damage variable evolution for various finite elements. The damage variable is plotted against the time (i.e., load step number). The damage distribution in the computational domain is not uniform. Since the damage variable is calculated based on input from the stress and strain distributions, damage localization should be expected in regions with stress and strain concentrations. While Figure 7 outlines the effect of self-healing by presenting the damage evolution in a single finite element, Figure 12 presents the complexity of the response of a specimen made of damageable material with healing capabilities.

In the pre-loading phase, damage accumulation starts simultaneously in finite elements A, E, and F in load step (LS) 10. In finite elements B, C, and D, the damage onset is delayed: it occurs later, in LS 18. At the end of the pre-loading phase, the maximum damage level is reached in finite element A. Comparing the damage evolution in finite elements D, E, and F, it can be seen that damage in D, although initiated later, accumulates faster and at the beginning of the healing process is greater than that in E and F. When healing is triggered, finite element C is fully healed, and no significant damage is detected in this finite element in the post-healing phase. In all other monitored finite elements, residual damage is apparent, after the healing process is accomplished. In the post-healing phase, the residual damage is maintained constant until the accumulation starts accumulating again in LS 40 for finite element A, in LS 43 for B and D, and in LS 44 for E and F. The critical value of the damage variable, $D_{c}$, is reached at the earliest in finite element A in LS 41, and later in B and D in LS 43. Damage accumulation in E and F is also reinitiated in the post-healing phase, but without reaching $D_{c}$.

**Figure 13:**
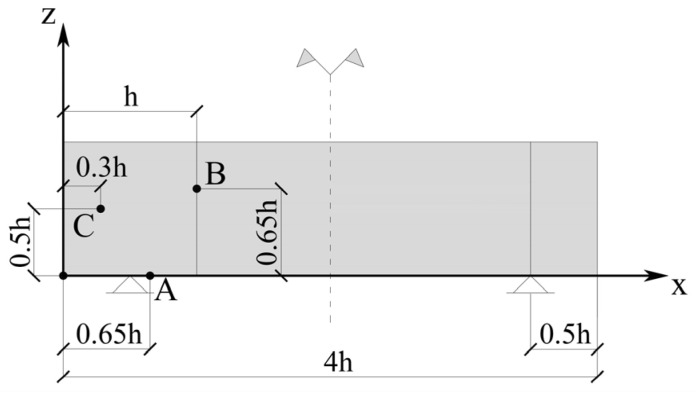
Positions of the monitored locations.

Positions A, B, and C (Figure 12a) are displayed in Figure 13.

Before cracking, damage accumulation is best pronounced in position A (compared to positions B and C), since on the bottom surface between the supports, the tensile stress and strain components are larger. Therefore, damage accumulation at location A (in the pre-loading phase) starts earlier and evolves faster than in the other two locations, as shown in Figure 12a. Damage accumulation at location B starts later than that at location A, given that location B is situated relatively far from the initial tensile zone of the specimen subjected to four-point bending. The onset of damage at location C is correlated with progressive damage and cracking in the tensile zone between the supports, provoking changes in the initial characteristics of the specimen’s cross-section. As expected, after self-healing triggering, damage at location C remains insignificant.

Tracking the local damage evolution can be used to determine the most effective placement of the self-healing agent and accurately define the optimal moment to trigger healing by simulating various scenarios. Such visualization can also have potential applications to maintenance planning of structures, if repeated self-healing interventions are required during the exploitation period. The latter situation remains out of the scope of this article.

## 4. Discussion

Table 5 summarizes some models discussed in Section 1.

It can be seen that all the above damage-healing models are either calibrated for materials other than cementitious composites or focused on purely theoretical examples.

Unlike most algorithms that reproduce the behavior of materials exhibiting both damageable and healing responses, the approach proposed in this article employs a single damage variable. This choice appears justified, given that self-healing can be a highly user-controlled process (for a triggering different from the mechanical one), rather than a result of the interaction of several natural mechanisms (for example, during the resting and unloading periods of cyclic loading applied to bituminous materials). Therefore, the introduction to various constitutive relationships describing different coupled natural phenomena is considered not mandatory. The applied mechanical load provokes damage accumulation in the material, which is accounted for (in the model) by the damage variable increase and the corresponding degradation of mechanical properties. The damage variable is also modified once the healing process is initiated; it is reset to zero or substantially decreased, depending on the type and amount of the healing agent.

Although the numerical examples illustrate only a single-step treatment procedure, the algorithm does not imply restrictions on the number of activations. It should be noted that the loading history is also accounted for by tracking and recording the damage variable and material properties throughout the solution.

The reported results are obtained by simulating characterization tests in which the material is mostly subjected to a unidirectional stress state. However, a 3-D finite element setting is employed, and the implemented solution algorithm is also three-dimensional. The output can thus be regarded as validation before implementing the algorithm in more complex case studies, supposing multiaxial loading.

The results obtained by finite-element simulations demonstrate the ability of the employed algorithm to analyze the damage and the healing response of concrete structural members. The time for curing is present yet implicitly in the proposed approach. The definition of an explicit time-dependent healing function is forthcoming.

Every model requires a calibration based on experimental input. For example, the model reported herein implies tuning of the healing efficiency parameter and, more precisely, the proper manipulation of the damage variable, based on experimental data. In this context, a model that investigates the phenomena within the RVE would provide an accurate assessment of the healing variable. Formulation of a model representing the microcapsules, investigating the conditions under which the shells of the latter break and release the healing agent, the spreading of the healing agent into the formed cracks, and studying the overall response of the RVE containing cracks filled with a polymerized healing agent, is underway.

## 5. Conclusions

A numerical algorithm employing a constitutive relationship rooted in continuum damage mechanics has been implemented to analyze the damage-healing response of concrete specimens with polymeric self-healing microcapsules.

Numerical simulations have been run to gain insight into the behavior of specimens subjected to compression and tension, specifically, tension by flexure, for the macroscopic characterization of both concrete (or the cementitious material) and the healing agent efficiency.

The obtained numerical results show that recovery of compressive strength becomes more pronounced for higher concrete grades and increases with the increase in the healing efficiency $\left(p\right)$, within the considered range. For a 20% increase in p, the strength recovery $\left(I_{R}^{\left(c\right)}\right)$ increase is 18.5%, 32%, and 53.2% for C25, C30, and C35, respectively. On the contrary, simulations predict a minor influence of the concrete grade for specimens subjected to tension by flexure. For the same increase in p, the strength recovery $\left(I_{R}^{\left(t\right)}\right)$ increaseis 3.5%, 5.3%, and 4.6%, for C25, C30, and C35, respectively.

Additionally, a study of local behavior is performed through element-wise tracking of the damage evolution. It can be regarded as a tool for informed decisions about the placement of the polymeric microcapsules and the scheduling of the optimal triggering moment.

Forthcoming related research includes:

- Development and finite-element implementation of a constitutive relationship accounting for the damage-induced anisotropy within the RVE through appropriate modification of the rigidity tensor.
- Modeling within the RVE, including an explicit definition of microcapsules with their shells, degradable interfaces, developed cracks, healing agent release, transport mechanisms, polymerized healing agent description, etc., for the rational quantification of the damaged-healed state, as an alternative to the empirical identification of the healing efficiency parameter.
- Application of the constitutive relationships for damage-healing materials in the analysis of the response of full-scale members. Combined with a systems-level study, self-healing can improve the fragility curves of a structure.

## Figures and Tables

**Table 1:** Mesh-sensitivity study.

Set	Maximum Finite Element Size	Number of Generated Finite Elements	Similarity Index, I_s_ (%)
1	23	1050	Reference
2	20	1456	0.993
3	17	2147	0.987
4	14	3220	0.984

**Table 2:** Quantitative assessment of self-healing for specimens loaded in compression.

C25		C30		C35	
$D_{max}^{\left(p\right)}=0.38$		$D_{max}^{\left(p\right)}=0.41$		$D_{max}^{\left(p\right)}=0.42$	
p	$I_{R}^{\left(c\right)}\left(\%\right)$	p	$I_{R}^{\left(c\right)}\left(\%\right)$	p	$I_{R}^{\left(c\right)}\left(\%\right)$
0.3	26.1	0.3	30	0.3	38.5
0.5	44.6	0.5	62	0.4	60.5
0.7	79.6	0.6	95.2	0.5	91.7

**Table 3:** Identification of the healing efficiency parameter p.

#	*p*	$I_{R}^{\left(c\right)}\left(\%\right)$	Reconstruction Error
1	0.15	42.2	108.1
2	0.195	67.5	30.1
3	0.2	70.9	23.8
4	0.205	74.4	18.0
5	0.22	85.5	2.7
6	0.25	102.3	-

**Table 4:** Quantitative assessment of self-healing efficiency for specimens loaded in tension by flexure.

C25		C30		C35	
p	$I_{R}^{\left(t\right)}\;\left(\%\right)$	p	$I_{R}^{\left(t\right)}\;\left(\%\right)$	p	$I_{R}^{\left(t\right)}\;\left(\%\right)$
0.3	91.7	0.3	88.8	0.3	89.7
0.4	93.6	0.4	91.7	0.4	92.1
0.5	95.2	0.5	94.1	0.5	94.3

**Table 5:** Digest of the damage-healing models.

Author	Scale	Damage Variables	Case Studied
Mergheim and Steinmann, 2013 [31]	macroscopic	Scalar damage and healing variables	1-D examples for thermosetting materials
Barbero et al., 2005 [32]	macroscopic	Damage- and healing variable: second rank tensors	Self-healing composites; Effect of healing on Carbon-Epoxy T300-5208; The in-plane shear stress–strain curves demonstrate the increased shear stiffness and strength due to the self-healing action.
Voyiadjis et al., 2012 [33]	macroscopic	Second rank anisotropic damage and healing variable tensors Fourth-rank anisotropic damage and healing variable tensors	Captures the irregular inelastic deformation of SMP and PET; Plastic and damage response of glassy polymers; Study the response of SMP-based self- healing system.
Al-Rub et al., 2010 [36]	Macroscopic/ microscopic	Microdamage healing internal state variable	Prediction of highly nonlinear mechanical response of asphalt mixture.
Pan et al., 2018 [37]	Macroscopic	Scalar damage and healing variables	Healing of cutting and puncture damage; Homogeneous healing under uniaxial tensile stress and healing under pure bending.

## Data Availability

All results presented and discussed in the article are obtained by finite element analyses performed by the author. No other data is employed.

## References

[1] Ferrara L., Van Mullem T., Alonso M.C., Antonaci P., Borg R.P., Cuenca E., Jefferson A., Ng P.-L., Peled A., Roig-Flores M. (2018). Experimental characterization of the self-healing capacity of cement based materials and its effects on the material performance: A state of the art report by COST Action SARCOS WG2. Constr. Build. Mater..

[2] Mihashi H., Nishiwaki T. (2012). Development of engineered self-healing and self-repairing concrete-state-of-the-art report. J. Adv. Concr. Technol..

[3] Edvardsen C. (1999). Water Permeability and Autogenous Healing of Cracks in Concrete. ACI Mater. J..

[4] Huang H., Ye G. (2012). Simulation of self-healing by further hydration in cementitious materials. Cem. Concr. Compos..

[5] Granger S., Loukili A., Pijaudier-Cabot G., Chanvillard G. (2007). Experimental characterization of the self-healing of cracks in an ultra high performance cementitious material: Mechanical tests and acoustic emission analysis. Cem. Concr. Res..

[6] Zhang L., Zheng M., Zhao D., Feng Y. (2024). A review of novel self-healing concrete technologies. J. Build. Eng..

[7] White S.R., Sottos N.R., Geubelle P.H., Moore J.S., Kessler M.R., Sriram S.R., Brown E.N., Viswanathan S. (2001). Autonomic healing of polymer composites. Nature.

[8] Ren J., Wang X., Li D., Xu S., Dong B., Xing F. (2021). Performance of temperature adaptive microcapsules in self-healing cementitious materials under different mixing temperatures. Constr. Build. Mater..

[9] Huang W., Zhang J., Wang J., Zheng Y., Ma J., Ding F. (2023). Performance analysis of paraffin microcapsules and phase change concrete based on microporous cenospheres. Constr. Build. Mater..

[10] Dong B., Diao H., Ren H., Hong S., Wang Y., Fang G., Zhang Y. (2023). Chloride-ion-triggered microcapsule for self-suppression of capillary suction in cement paste. Cem. Concr. Compos..

[11] Meng F., Dong L., Wu Y., Shu X., Guo Y., Ran Q. (2023). Effects and mechanisms of capric acid/silica capsule on water absorption reduction of cement paste. Constr. Build. Mater..

[12] Song Z., Huang Z., Jia Z., Jiang L., Chu H., Zhang Y. (2024). Design of a hydrophobic nano-SiO_2_-modified ER@ EC microcapsule: Improving rheology, regulating hydration while preserving self-healing in cementitious materials. Cem. Concr. Compos..

[13] Lv L., Zhang X., Šavija B., Zhang M., Han K., Zhang H., Pei C., Zhu J., Xing F. (2024). Self-healing of cementitious materials using sustainable cenosphere-based manufactured aggregate. Constr. Build. Mater..

[14] Ying Y., Hu M., Han J., Liu W., Qi B., Guo J. (2023). Self-healing in cementitious system using interface enhanced capsules prepared at room temperature. J. Clean. Prod..

[15] Xu N., Song Z., Guo M.Z., Jiang L., Chu H., Pei C., Yu P., Liu Q., Li Z. (2021). Employing ultrasonic wave as a novel trigger of microcapsule self-healing cementitious materials. Cem. Concr. Compos..

[16] Wang S., Song Z., Chu H., Jiang L., Zhang Y. (2025). Design of an iron-flake-modified ER@EC microcapsule for ultrasound-triggered self-healing in cementitious materials. Cem. Concr. Compos..

[17] Li Y., Yu J., Cao Z., Du W., Zhang Y., Zou Y. (2020). Preparation and characterization of nano-Fe_3_O_4_/paraffin encapsulated isocyanate microcapsule by electromagnetic controlled rupture for self-healing cementitious materials. Constr. Build. Mater..

[18] Li Y., Yu J., Cao Z., He P., Liu Q., Han X., Wan Y. (2021). Preparation and application of novel microcapsules ruptured by microwave for self-healing concrete. Constr. Build. Mater..

[19] Chang H., Chen L., Guo F., Wang X., Li C., Li Z. (2025). Mechanical performance of concrete blended with high-strength capsules prepared by a novel method. J. Build. Eng..

[20] Riordan C., Anglani G., Inserra B., Palmer D., Al-Tabbaa A., Tulliani J.M., Antonaci P. (2023). Novel production of macrocapsules for self-sealing mortar specimens using stereolithographic 3D printers. Cem. Concr. Compos..

[21] Sinha A., Wang Q., Wei J. (2021). Feasibility and compatibility of a biomass capsule system in self-healing concrete. Materials.

[22] Sinha A., Lim D.Z.H., Wei J. (2022). A lignin-based capsule system with tunable properties tailored for robust self-healing concrete. Cem. Concr. Compos..

[23] Lim T., Cheng H., Hu J., Lee Y., Kim S., Kim J., Jung W. (2023). Development of 3D-printed self-healing capsules with a separate membrane and investigation of mechanical properties for improving fracture strength. Materials.

[24] Tsangouri E., Van Loo C., Shields Y., De Belie N., Van Tittelboom K., Aggelis D.G. (2022). Reservoir-vascular tubes network for self-healing concrete: Performance analysis by acoustic emission, digital image correlation and ultrasound velocity. Appl. Sci..

[25] Rosewitz J.A., Wang S., Scarlata S.F., Rahbar N. (2021). An enzymatic self-healing cementitious material. Appl. Mater. Today.

[26] Kachanov L. (1958). On the creep fracture time. Izv. Akad. Nauk SSSR Otd. Teckhnicheskikh Nauk..

[27] Rabotnov Y., Hetenyi M., Vincenti W.G. (1969). Creep rupture. Proceedings of the Twelfth International Congress of Applied Mechanics, Stanford, CA, USA, 26–31 August 1968.

[28] Lemaitre J. (1985). A continuous damage mechanics model for ductile fracture. ASME J. Eng. Mat. Technol..

[29] Cordebois J.P., Sidoroff F. (1982). Endommagement anisotrope en élasticité et plasticité. J. De Mécanique Théorique Et. Appliquée.

[30] Chaboche J.L. (1978). Description Thermodynamique et Phénoménologique de la Viscoélasticité Cyclique avec Endommagement. Ph.D. Thesis.

[31] Mergheim J., Steinmann P. (2013). Phenomenological modelling of self-healing polymers based on integrated healing agents. Comput. Mech..

[32] Barbero E.J., Greco F., Lonetti P. (2005). Continuum damage-healing mechanics with application to self-healing composites. Int. J. Damage Mech..

[33] Voyiadjis G.Z., Shojaei A., Li G., Kattan P.I. (2012). A theory of anisotropic healing and damage mechanics of materials. Proceedings of the Royal Society A: Mathematical. Phys. Eng. Sci..

[34] Hansen N.R., Schreyer H.L. (1994). A thermodynamically consistent framework for theories of elastoplasticity coupled with damage. Int. J. Solids Struct..

[35] Voyiadjis G.Z., Kattan P.I. (2009). A comparative study of damage variables in continuum damage mechanics. Int. J. Damage Mech..

[36] Al-Rub R.K.A., Darabi M.K., Little D.N., Masad E.A. (2010). A micro-damage healing model that improves prediction of fatigue life in asphalt mixes. Int. J. Eng. Sci..

[37] Pan Y., Tian F., Zhong Z. (2018). A continuum damage-healing model of healing agents based self-healing materials. Int. J. Damage Mech..

[38] Voyiadjis G.Z., Kattan P.I. (2017). Mechanics of damage, healing, damageability, and integrity of materials: A conceptual framework. Int. J. Damage Mech..

[39] Voyiadjis G.Z., Kattan P.I. (2024). A new unsymmetrical decomposition of the damage variable. Int. J. Damage Mech..

[40] Coleman B.D., Gurtin M.E. (1967). Thermodynamics with internal state variables. J. Chem. Phys..

[41] Lubliner J. (1972). On the thermodynamic foundations of non-linear solid mechanics. Int. J. Non-Linear Mech..

[42] Voyiadjis G.Z., Shojaei A., Li G. (2011). A thermodynamic consistent damage and healing model for self healing materials. Int. J. Plast..

[43] Al-Rub R.K.A., Darabi M.K. (2012). A thermodynamic framework for constitutive modeling of time- and rate-dependent materials. Part I: Theory. Int. J. Plast..

[44] Darabi M.K., Al-Rub R.K.A., Little D.N. (2012). A continuum damage mechanics framework for modeling micro-damage healing. Int. J. Solids Struct..

[45] Darabi M.K., Al-Rub R.K.A., Masad E.A., Little D.N. (2012). A thermodynamic framework for constitutive modeling of time-and rate-dependent materials. Part II: Numerical aspects and application to asphalt concrete. Int. J. Plast..

[46] Alsheghri A.A., Al-Rub R.K.A. (2016). Finite element implementation and application of a cohesive zone damage-healing model for self-healing materials. Eng. Fract. Mech..

[47] Reda M.A., Chidiac S.E. (2022). Performance of Capsules in Self-Healing Cementitious Material. Materials.

[48] Vedrtnam A., Kalauni K., Palou M.T. (2025). Finite element simulation of bacterial self-healing in concrete using microstructural transport and precipitation modeling. Sci. Rep..

[49] Zhelyazov T. (2020). Structural materials: Identification of the constitutive models and assessment of the material response in structural elements strengthened with externally-bonded composite material. Materials.

[50] Zhelyazov T. (2022). Numerical simulation of the response of concrete structural elements containing a self-healing agent. Materials.

[51] FIB—Fédération International du Béton (2013). FIB Model Code for Concrete Structures.

[52] Mazars J., Hamon F., Grange S. (2015). A new 3D damage model for concrete under monotonic, cyclic and dynamic loadings. Mater. Struct..

[53] Mazars J. (1986). A description of micro-and macroscale damage of concrete structures. Eng. Fract. Mech..

[54] Mazars J. (1984). Damage Mechanics Application to Nonlinear Response and Failure Behavior of Structural Concrete. Doctoral Dissertation.

[55] Chaboche J. (1988). Continuum damage mechanics. I: General concepts. J. Appl. Mech..

[56] Eem S.H., Jung H.J., Koo J.H. (2012). Modeling of magneto-rheological elastomers for harmonic shear deformation. IEEE Trans. Magn..

[57] Du W., Yu J., He B., He Y., He P., Li Y., Liu Q. (2020). Preparation and characterization of nano-SiO_2_/paraffin/PE wax composite shell microcapsules containing TDI for self-healing of cementitious materials. Constr. Build. Mater..

[58] Mura T. (1987). Micromechanics of Defects in Solids.

[59] Lv L., Schlangen E., Yang Z., Xing F. (2016). Micromechanical properties of a new polymeric microcapsule for self-healing cementitious materials. Materials.

